# Supplementary material for: The contrasting effects of body image and self-esteem in the makeup usage
Source: PLoS One. 2022 Mar 25;17(3):e0265197. doi: 10.1371/journal.pone.0265197 (PMC8956164; doi:10.1371/journal.pone.0265197)
Supplement: S1 File — (DOCX) [file pone.0265197.s001.docx]

S1 File. Additional analyses

| **S1 Table. Kendall correlations among the variables of frequency of makeup usage** | | | | | | |
| --- | --- | --- | --- | --- | --- | --- |
|  | | **Base, concealer, and/or powder** | **Mascara** | **Eyeliner or eye pencil** | **Shade** | **Lipstick and/or gloss** |
| **Base, concealer, and/or powder** | [τ](https://pt.wikipedia.org/wiki/%CE%A4) |  | ,482** | .279** | .350** | .327** |
|  | *p* |  | ≤.001 | ≤.001 | ≤.001 | ≤.001 |
| **Mascara** | [τ](https://pt.wikipedia.org/wiki/%CE%A4) | .482** |  | .354** | .338** | .318** |
|  | *p* | ≤.001 |  | ≤.001 | ≤.001 | ≤.001 |
| **Eyeliner or eye pencil** | [τ](https://pt.wikipedia.org/wiki/%CE%A4) | .279** | .354** |  | .397** | .294** |
|  | *p* | ≤.001 | ≤.001 | ≤.001 | ≤.001 | ≤.001 |
| **Shade** | [τ](https://pt.wikipedia.org/wiki/%CE%A4) | .350** | .338** | .397** |  | .300** |
|  | *p* | ≤.001 | ≤.001 | ≤.001 |  | ≤.001 |
| **Lipstick and/or gloss** | [τ](https://pt.wikipedia.org/wiki/%CE%A4) | .327** | .318** | .294** | .300** |  |
|  | *p* | ≤.001 | ≤.001 | ≤.001 | ≤.001 |  |
| ** Correlation is significant at the 0.01 level (2-tailed). | | | |  |  |  |

| S2 Table. Descriptive statistics | | | | | | | | | |  |  |  |
| --- | --- | --- | --- | --- | --- | --- | --- | --- | --- | --- | --- | --- |
|  | **N** | **Minimum** | **Maximum** | **Mean** | **Standard Deviation** | **Skewness** | | **Kurtosis** | | **Kolmogorov-Smirnov^a^** | | |
|  |  |  |  |  |  | **Stats** | **Standard model** | **Stats** | **Standard model** | **Stats** | **df** | **p** |
| Age | 1483 | 18 | 75 | 31.0809 | 11.1515 | 1.166 | 0.064 | 1.036 | 0.127 | 0.115 | 985 | 0 |
| Family income | 1405 | 1 | 8 | 4.96 | 1.722 | 0.399 | 0.065 | -0.732 | 0.13 | 0.176 | 985 | 0 |
| Spent value on makeup per month | 1381 | 1 | 9 | 3.4 | 2.066 | 0.768 | 0.066 | -0.188 | 0.132 | 0.17 | 985 | 0 |
| Spent time on makeup per day | 1381 | 1 | 5 | 1.85 | 0.972 | 1.076 | 0.066 | 0.652 | 0.132 | 0.269 | 985 | 0 |
| Makeup usage frequency | 1288 | 1 | 7 | 3.9548 | 135.207 | 0.184 | 0.068 | -0.704 | 0.136 | 0.061 | 985 | 0 |
| Appearance orientation | 1088 | 1.67 | 5 | 3.6426 | 0.63108 | -0.189 | 0.074 | -0.292 | 0.148 | 0.039 | 985 | 0.001 |
| Appearance evaluation | 1086 | 1 | 5 | 3.246 | 0.91514 | -0.402 | 0.074 | -0.513 | 0.148 | 0.092 | 985 | 0 |
| Personal self-esteem | 1222 | 10 | 40 | 29.725 | 6.22718 | -0.396 | 0.07 | -0.417 | 0.14 | 0.048 | 985 | 0 |
| Social Self-esteem | 1078 | 1.2 | 6 | 4.1717 | 0.91994 | -0.282 | 0.075 | -0.347 | 0.149 | 0.072 | 985 | 0 |

**S3 Table. Frequency of spent value on makeup per month**

|  | Frequency | Percentage |  |  |
| --- | --- | --- | --- | --- |
| Zero | 286 | 20.7 |  |  |
| Less than R$10 | 274 | 19.8 |  |  |
| From R$11 to R$25 | 255 | 18.5 |  |  |
| From R$26 to R$40 | 196 | 14.2 |  |  |
| From R$41 to R$60 | 145 | 10.5 |  |  |
| From R$61 to R$80 | 79 | 5.7 |  |  |
| From R$81 to R$100 | 74 | 5.4 |  |  |
| From R$101 to R$200 | 52 | 3.8 |  |  |
| More than R$200 | 20 | 1.4 |  |  |
| Total | 1381 | 100 |  |  |

**S4 Table. Frequency of spent time on makeup per day**

|  | Frequency | Percentage |
| --- | --- | --- |
| Less than 5 minutes | 628 | 45.5 |
| From 5 to 10 minutes | 447 | 32.4 |
| From 10 to 20 minutes | 211 | 15.3 |
| From 20 to 30 minutes | 72 | 5.2 |
| More than 30 minutes | 23 | 1.7 |
| Total | 1381 | 100 |

**S5 Table. Frequency of makeup usage**

|  | **Base, concealer, and/or powder** | | **Mascara** | | **Eyeliner or eye pencil** | | **Shade** | | **Lipstick and/or gloss** | | **Frequency of makeup usage (total)** | | |
| --- | --- | --- | --- | --- | --- | --- | --- | --- | --- | --- | --- | --- | --- |
|  | **Frequency** | **Percentage** | **Frequency** | **Percentage** | **Frequency** | **Percentage** | **Frequency** | **Percentage** | **Frequency** | **Percentage** | **Frequency** | **Percentage** |  |
| **Never** | 92 | 6.8 | 65 | 4.9 | 148 | 11.2 | 208 | 15.8 | 34 | 2.5 | 22 | 1.7 |  |
| **Rarely** | 236 | 17.5 | 184 | 14.0 | 363 | 27.4 | 518 | 39.3 | 138 | 10.3 | 178 | 13.8 |  |
| **Sometimes** | 317 | 23.6 | 313 | 23.8 | 368 | 27.8 | 356 | 27.0 | 261 | 19.6 | 317 | 24.6 |  |
| **Half of the time** | 61 | 4.5 | 87 | 6.6 | 70 | 5.3 | 52 | 3.9 | 102 | 7.6 | 320 | 24.8 |  |
| **Frequently** | 185 | 13.7 | 186 | 14.2 | 115 | 8.7 | 65 | 4.9 | 221 | 16.6 | 263 | 20.4 |  |
| **Most of the times** | 149 | 11.1 | 132 | 10.0 | 93 | 7.0 | 59 | 4.5 | 155 | 11.6 | 145 | 11.3 |  |
| **Always** | 306 | 22.7 | 347 | 26.4 | 166 | 12.5 | 60 | 4.6 | 423 | 31.7 | 43 | 3.3 |  |
| **Total** | 1346 | 100 | 1314 | 100 | 1323 | 100 | 1318 | 100 | 1334 | 100 | 1288 | 100 |  |
